# Status and related factors of medication safety behaviour of nurses in the operating room: A cross-sectional survey in China

**DOI:** 10.1371/journal.pone.0320264

**Published:** 2025-04-28

**Authors:** Xiuwen Chen, Xueyi Wei, Liqing Yue, Duo Wu, Jiqun He

**Affiliations:** 1 Xiangya School of Nursing, Central South University, Changsha, China; 2 Teaching and Research Section of Clinical Nursing, Xiangya Hospital, Central South University, Changsha, China; 3 National Clinical Research Center for Geriatric Disorders, Xiangya Hospital, Central South University, Changsha, China; 4 Department of Nursing, The Xiangya Hospital, Central South University, Changsha, China; The World Islamic Sciences and Education University, JORDAN

## Abstract

**Aim:**

This study aimed to investigate the current status and influencing factors of medication safety behaviour of operating room nurses.

**Background:**

Medication safety is an important safety issue recognised by health organisations all over the world, and the operating room has one of the highest rates of preventable medication-related injuries. However, few studies have investigated the current status and influencing factors of medication safety behaviour of operating room nurses.

**Design:**

A cross-sectional study.

**Methods:**

From February to March, this study was conducted at three tertiary hospitals in southern China. Operating room nurses completed a series of questionnaires, including nurses’ medication environment perception scale and operating room nurse’s medication safety behaviour questionnaire. Data analysis included descriptive statistics, ANOVA, correlation analysis, and multiple regression. The STROBE checklist guided the reporting of this study.

**Results:**

A total of 171 questionnaires were analysed. The total score on medication safety behaviour of operating room nurses was 78.20±8.94. The medication environment the operating room nurses perceived was positively correlated with medication safety behaviour (*P* < 0.01). Additionally, factors related to the medication safety behaviour of operating room nurses included working years(*B* = 4.899, *P* = 0.000), the highest level of education(*B* = 5.440, *P* = 0.000), professional title(*B* = −2.644, *P* = 0.002), the last time of nursing medication safety training(*B* = −0.914, *P* = 0.013), and the *system and supervision* (*B* = 0.141, *P* = 0.015).

**Conclusion:**

The medication safety behaviour of operating room nurses is low. The relationship between individual factors, medication environment, and medication behaviour of operating room nurses should be deeply considered, and targeted intervention strategies should be carried out to influence factors to improve their medication safety behaviour.

## 1 Introduction

The World Health Organization (WHO) launched the global patient safety challenge on medication safety in 2017, which aimed to reduce serious and avoidable medication-related harm by 50% in all countries over the next five years by addressing the weaknesses in health systems that lead to medication errors and the severe harm that results [1]. Medication safety is an important safety issue recognised by health organisations worldwide [2]. Medication administration errors (MAEs) refer to drug administration errors caused by deviation from the doctor’s order or prescription content in the case of correct prescription [3]. Studies have shown that the incidence of MAEs is about 10%, ranging from approximately 5% to 20% of medication administrations in hospitals, and MAEs are the leading cause of patient harm [4]. Costs associated with medication errors are estimated at $42 billion annually globally [5]. MAEs may occur at all stages of drug administration, and unsafe medication behaviour of clinical nurses is the main factor leading to MAEs [6,7]. Therefore, improving the ability of nurses to safely administer medication is crucial to achieving this initiative.

The operating room (OR) is the setting for the most invasive treatments in the hospital, where high-risk drugs such as narcotic, toxic, and powerful drugs are routinely administered, and the incorrect selection or improper use of high-risk drugs is likely to result in serious accidents [8]. Most surgical patients cannot express any discomfort following medication administration because they are sedated during the operation, making the consequences of medication misuse particularly severe. The Institute for Safe Medication Practices (ISMP) has reported serious medical incidents resulting in severe burns and even death due to intraoperative medication errors [9]. Most studies have found that intraoperative medication errors pose potential threats to patient safety and even endanger patients’ lives, and failure to implement safety strategies will cause such risks to persist for a long time [10,11]. In addition, the high-pressure, fast-paced, and tense working environment in the operating room, coupled with frequent oral medical instructions, increases the likelihood of medication errors [12]. A prospective observational study of medication errors in OR showed that 193 out of 3 671 MAEs occurred in OR, with an incidence of 5.3%, and 79.3% were preventable [13]. Additionally, studies have pointed out that one medication administration error occurs in every one operation or every 20 dosing, and nearly one-third of MAEs will cause harm to patients [12,14]. Despite this, the operating room remains one of the areas with the highest rate of preventable medication-related injuries [15]. This highlights the need to pay attention to medication safety in the operating room to reduce or eliminate medication errors.

Operating room nurses play a crucial role in drug administration, including dispensing, checking, and assisting the anesthesiologist in administering drugs [8]. They are not only the observers of the reaction after drug administration but also the implementors and protectors of safe drug use, shouldering important responsibilities in ensuring drug safety and also the last line of defence to eliminate medication errors in the operating room [16]. Their behaviour is directly related to the occurrence of MAEs. However, in recent years, studies on medication safety in OR have mainly focused on investigating incidence, monitoring medication errors, reporting methods for MAEs, and evaluating the effectiveness of quality management [17–19]. There have been few studies investigating the medication safety behaviour of OR nurses. Therefore, this study aimed to explore the status and related factors of medication safety behaviour of operating room nurses and to provide a basis for the formulation of medication risk management measures in OR.

## 2 Methods

### 2.1 Study design and participants

From February to March 2023, a cluster random sampling method was used to randomly select three tertiary hospitals in southern China. Specifically, the list of all three tertiary hospitals in southern China was compiled, and the hospitals were divided into several groups based on their geographical location and hospital scale. Next, the research team randomly selected three tertiary hospitals from the compiled list. Then, a cross-sectional study of operating room nurses from the selected three hospitals was conducted to investigate their medication safety behaviour. The Guidelines for the Strengthening the Reporting of Observational Studies in Epidemiology(STROBE) were used in this study [20].

The inclusion criteria are as follows: (1)having a nurse license; (2) having at least 1 year of clinical nursing experience; (3) working in the operating room; and (4) informed consent and voluntary participation in the study. Exclusion criteria: (1) nurse trainee; (2)unable to participate due to physical illness; and (3) nurses not on duty or vacation.

For the sample size, the recommendations are between 5 and 10 times the number of explanatory variables [21,22]. With an estimated 15 explanatory variables (10 demographic variables, five dimensions of nurses’ medication environment perception scale) and accounting for 20% of invalid questionnaires, a minimum of 90 operating nurses was required.

### 2.2 Measurements

#### 2.2.1 Demographic information questionnaire.

According to the purpose of the research, the general information questionnaire was made, and the participants’ demographic information included age, sex, the highest level of education, present position, professional title, working years, marital status, average monthly income, experience of medication safety training and the last time of nursing medication safety training.

#### 2.2.2 Nurses’ medication environment perception scale.

The scale was compiled by Liu et al. [10]. based on the SHEL (Software, Hardware, Environment, Liveware) model to screen nurses’ perception of the medication environment. The scale includes 32 items across five dimensions: *System and supervision* (11 items), *equipment* (4 items), *environment* (3 items), *nurses* (7 items), and *relevant personnel* (7 items). The *system and supervision* dimension refers to the perception of safety regarding rules, regulations, and management processes involved in the process of nurses’ medication. The dimensions of *environment* and *equipment* refer to the safety perception of nurses’ working environment and equipment used in the medication process, respectively. The dimension of *a nurse refers to the perception of safety regarding nurses’ professional qualities*. The *relevant personnel* dimension refers to the safety perception of other personnel (doctors, patients) related to the medication nurse. All items are scored on a five-point Likert-like scale, ranging from “strongly disagree” to “strongly agree”, with a total score ranging from 32 to 160 points. The higher the score, the better the medication environment perceived. The Cronbach’s alpha of the scale in this study was 0.979.

#### 2.2.3 Operating room nurses medication safety behavior questionnaire.

The questionnaire was designed by the research group based on the previous research and modified, discussed, and compiled by the group on the basis of extensive references to relevant literature. There are 28 items; the first two are true or false questions, “Yes” counts 1 point, and “No” counts 5 points. The last 26 questions are scored on a five-point Likert-like scale, with “never”, “rarely”, “sometimes”, “often” and “always”. The questionnaire is divided into five dimensions, including *medication placement* (4 items), *doctor’s order and check* (8 items), *dispensing* (6 items), *aseptic principle*(3 items), *inspection and transport*(5 items), with a total score ranging from 28 to 140 points. The higher the score, the higher the medication safety behaviour of the operating room nurses. The Cronbach’s alpha of the questionnaire was 0.678.

### 2.3 Data collection

Electronic questionnaires were used to collect data. The researchers first contacted the head nurses in the operating rooms of three randomly selected hospitals and briefly trained them on the methods of data collection and precautions. The questionnaire link was sent to 3 head nurses, and the head nurses sent the link to the nurses who met the inclusion and exclusion criteria. The questionnaire used unified guidance to explain the purpose of the survey, answer methods, filling requirements and precautions, etc. The results were directly returned to the researchers after the operating room nurses completed the questionnaire. To ensure the quality of the survey results, all researchers received uniform and pre-survey training, and researchers worked rigorously and communicated skillfully. Re-enter and check the data after collecting the questionnaire to ensure its authenticity and reliability.

### 2.4 Statistical analysis

Excel was used for data entry, and IBM SPSS v25.0 was used for data analysis. The measurement data were described by means and standard deviations, while the count data were expressed as frequencies and percentages. Statistical inference was carried out using the test level of α = 0.05. Bilateral probability values were taken as *P*-values. *T*-test and ANOVA were used for data with normal distribution and homogeneity of variance. Pearson correlation analysis was used for correlation analysis, and multiple linear regression was used for regression analysis. *p* < 0.05 was considered statistically significant. The scoring rate is the actual score (theoretical maximum(100%) [23]. The scoring rates greater than 80%, between 60% and 80%, and less than 60% indicate that the medication safety behaviour is good, medium and poor, respectively.

### 2.5 Ethical considerations

This study was approved by the Ethics Committee of Xiangya Hospital, Central South University (registry number 2022100085). The study complied with the Declaration of Helsinki; all participants signed informed consent forms and participated anonymously.

## 3 Results

### 3.1 Sociodemographic characteristics and univariate analysis of the study participants

A total of 174 questionnaires were distributed, while 171 questionnaires were valid, with an effective response rate of 98.3%. Among the participants, 27.5%, 22.2%, 34.5% and 15.8% worked for <6 years, 6–10 years, 11–20 years and >21 years, respectively. About 91.2% of the sample was female, and males accounted for 8.8%. 45.0% of nurses were aged 31–40, and 70.8% had bachelor’s degrees. The main technical titles were supervisor nurses, accounting for 46.8%. About 86.0% of nurses had received medication safety training. The normality test showed that all the demographic variables in this study obeyed normal distribution except the highest level of education and average monthly income. Analysis of variance, Kruskal-Wallis test and *t*-tes*t* revealed that the demographic factors related to the medication safety behaviour of OR nurses were age, working years, highest level of education, average monthly income, present position, professional title, experience of medication safety training and the last time of nursing medication safety training (*p* < 0.05). Other demographic details and univariate analysis results are shown in Table 1.

**Table 1:** Sample demographics and Univariate analysis (n = 171).

Variable	Category Group	Frequency (%)	Univariate analysis results
Age (years)	21–30	57(33.3)	*F* = 8.027 *P* = 0.000*
	31−40	77(45.0)	
	41–50	28(16.4)	
	51–60	9(5.3)	
Sex	Male	15(8.8)	*t* = −0.243 *P* = 0.808
	Female	156(91.2)	
Working years (years)	<6	47(27.5)	*F* = 13.983 *P* = 0.000*
	6–10	38(22.2)	
	11–20	59(34.5)	
	>21	27(15.8)	
Marital status	Married	120 (70.2)	*F* = 0.284 *P* = 0.753
	Spinsterhood	45(26.3)	
	others	6(3.5)	
Highest level of education	Associate degree	43(25.1)	*H* = 14.379 *P* = 0.001*
	Bachelor degree	121(70.8)	
	Master’s degree and above	7(4.1)	
Average monthly income(CNY)	<5000	32(18.7)	*H* = 30.814 *P* = 0.000*
	5000–10000	98(57.3)	
	>10000	41(24.0)	
Present position	Nurse	116(67.8)	*F* = 4.260 *P* = 0.006*
	Head nurse	31(18.1)	
	Specialist group leader	18(10.5)	
	Others	6(3.5)	
Professional title	Nurse	25(14.6)	*F* = 2.691 *P* = 0.033*
	Senior nurse	38(22.2)	
	Supervisor nurse	80(46.8)	
	Deputy chief nurse	27 (15.8)	
	Chief nurse	1(0.6)	
Experience in medication safety training	Yes	147(86.0)	*t* = −0.641 *P* = 0.522
	No	24(14.0)	
The last time nursing medication safety training	Within a month	45(30.6)	*F* = 2.683 *P* = 0.023*
	Within three months	41(27.9)	
	Within half a year	27(18.4)	
	Within a year	13(8.8)	
	More than a year	21(14.3)	

Note:

*indicates *P<*0.05; CNY stands for Chinese Yuan

### 3.2 Medication environment perception of operating room nurses

This study showed that operating room nurses’ total score of medication environment perception was 134.05±25.47, and the scoring rate was 83.8%. The scoring rate of each dimension from high to low was *system and supervision*, *nurses* and *environment, equipment*, and *related personnel.* Refer to Table 2 for the specific information of each dimension.

**Table 2:** Scores of medication environment perception of operating room nurses(n = 171).

Dimension	Score (Mean ± SD)	Scoring rate(%)
System and supervision	48.39±9.74	88.0
Nurses	30.78±6.02	87.9
Environment	13.18±2.69	87.9
Equipment	16.16±4.10	80.8
Related personnel	25.54±5.95	73.0

### 3.3 Medication safety behaviour of operating room nurses

The total score of medication safety behaviour of nurses in the OR was 78.20±8.94, and the scoring rate was 53.7%, which was moderate in *inspection and transport medication placement* but low in *doctor’s order and check*, *dispensing* and *aseptic principle*. In addition, item 1 and item 2 showed that 35 participants (20.46%) have experienced medication administration errors, and 11 nurses (6.43%) have experienced medication administration errors that have caused disputes. The detailed information on each dimension score is shown in Table 3. Additionally, the three items with the lowest score were Q12. First aid drugs will be carried during the skin test, and drugs requiring a skin test will be administered after the skin test (1.28±0.746), Q17. Will not be based on experience to prepare drugs without understanding drug compatibility contraindications (1.32±0.717), Q18 Will not be to complete work tasks or save time in advance to prepare drugs without considering the drug time limit required by the instructions(1.35±0.821).

**Table 3:** Scores of medication safety behaviour of operating room nurses(n = 171).

Dimension	Score (Mean ± SD)	Scoring rate(%)
Inspection and transport	16.15±2.47	64.6
Medication placement	12.73±2.39	63.7
Doctor’s order and check	22.35±3.15	55.9
Dispensing	12.51±3.27	41.7
Aseptic principle	5.53±2.38	36.9

### 3.4 Correlation analysis between medication environment perception and medication safety behaviour of operating room nurses

Pearson correlation analysis showed that the total score of operating room nurses’ perception of the medication environment was positively correlated with the total score of medication safety behaviour (*P* < 0.01). The correlation between each dimension of operating room nurses’ perception of the medication environment and each dimension of medication safety behaviour is shown in Table 4.

**Table 4:** Correlation analysis between medication environment perception and medication safety behaviour of operating room nurses (*r*-value).

Dimension	Medication placement	Doctor’s order and check	Dispensing	Aseptic principle	Inspection and transport	Total score of medication safety behavior
System and Supervision	0.270**	0.150	−0.083	−0.161*	0.468**	0.218**
Equipment	0.242**	0.003	−0.186*	−0.175*	0.453**	0.146
Environment	0.279**	0.093	−0.114	−0.216**	0.445**	0.187*
Nurses	0.270**	0.125	−0.122	−0.174*	0.484**	0.197**
Relevant personnel	0.189*	0.213**	0.052	0.005	0.317**	0.248**
The total score of medication environment precipitation	0.280**	0.147	−0.090	−0.152*	0.487**	0.231**

Note:

**indicates *P* < 0.01,

*indicates *P* < 0.05

### 3.5 Multivariate analysis of medication safety behaviour of nurses in the operating room

The total score of medication safety behaviour of operating room nurses was taken as the dependent variable, the demographic variables were statistically significant in the univariate analysis, and the five dimensions of the medication environment perception scale were taken as the independent variables for multivariate analysis by stepwise regression method (Assignment of independent variables was shown in Table 5). The results showed that working years, the highest level of education, professional title, the last time of nursing medication safety training, *and system and supervision* were the related factors of medication safety behaviour of operating room nurses. See Table 5 for details.

**Table 5:** Assignment of independent variables and multivariate analysis of medication safety behaviour of nurses in the operating room (n = 171).

Variable	*B*-Value	Standard Error	*t*	*P*	Assignment
Working years	4.899	0.753	6.502	0.000	“<6” = 1; “6–10” = 2; “11–20” = 3; “>21” = 4
Highest level of education	5.440	1.118	4.864	0.000	“Associate degree” = 1”; “Bachelor degree” = 2; “Master degree and above” = 3
Professional title	−2.644	0.852	−3.103	0.002	“Nurse” = 1; “Senior nurse” = 2; “Supervisor nurse” = 3; “Deputy chief nurse” = 4; “Chief nurse” = 5
The last time nursing medication safety training	−0.914	0.363	−2.522	0.013	“Within a month” = 1; “Within three months” = 2; “Within half a year” = 3; “Within a year” = 4; “More than a year” = 5
System and supervision	0.141	0.057	2.469	0.015	“strongly disagree” = 1; “disagree” = 2; “neutrality” = 3; “agree” = 4; “strongly agree” = 5

## 4 Discussion

To the best of our knowledge, this study clarifies the current state and related factors of medication safety behaviour of OR nurses, thereby contributing valuable insights to the existing body of knowledge on this topic. Our study reveals that the overall medication safety behaviour of OR nurses was low, with the medication environment perceived by the OR nurses emerging as a significant influencing factor. Additionally, working years, the highest level of education, system and supervision were positively correlated with the medication safety behaviour of OR nurses. Notably, their professional title and the last medication safety training were negatively correlated with their medication safety behaviour.

### 4.1 Medication safety behaviour of operating room nurses

The results of this study showed that the scoring rate of medication safety behaviour of OR nurses was at a low level, indicating that there are still certain limitations and deficiencies in the safe medication management process of OR nurses. To our knowledge, this is the first time such a study has been conducted, so it cannot be compared with previous findings. However, a previous study in Egypt that investigated the practice of general nurses in medication administration showed similar findings to this study [24]. In addition, A national survey of nurses in China showed a high proportion of unsafe medication behaviours among nurses [25]. Yu et al. [26] also showed that a considerable number of nurses experienced unsafe medication behaviours when administering drugs. However, the above research results are for reference only. The operating room environment’s particularity makes the nurses’ medication behaviour different from that of general nurses. In the future, a large-sample study should be carried out to investigate the medication behaviour of this specialised nurse. The OR is characterised by a large variety of high-risk drugs, high consumption, less management staff, less available storage space, and short drug preparation time etc, which makes the work of specialised nurses onerous [11]. Studies have shown that work pressure is one of the risk factors for nursing errors, and nurses in the operating room are under greater pressure than nurses in other departments [27].In addition, the intensive turnover of narcotic drugs and psychotropic drugs in the OR leads to a series of problems, such as difficult drug management and poor access to intraoperative drugs. According to the human error model developed by Professor Reason, unsafe medication behaviour is the direct cause of medication errors and may eventually lead to clinical adverse events [28]. Therefore, medication safety behaviour is one of the important indicators for managers to improve medication errors. Managers should strengthen operating room nurses’ management of unsafe medication behaviour, especially the management of unsafe behaviours that will not lead to medication errors, which is crucial for controlling medication errors.

### 4.2 Factors related to medication safety for operating room nurses

#### 4.2.1 The medication environment perceived by the OR nurses.

The medication environment can indirectly affect nurses’ medication safety behaviour by influencing their professional quality [29,30]. This study showed that the medication environment perceived by OR nurses positively correlated with medication safety behaviour, indicating that a good medication environment promotes nurses to perceive the hospital’s safety culture and improve their medication safety ability. The nurse’s work environment has long been recognised as an important and modifiable organisational feature that affects patient outcomes, encouraging nurses to think critically about medical treatment and sequences of care and make recommendations for care plans [31]. According to reciprocal determinism, the organisational environment is the basis of work behaviour and plays a guiding role in this behaviour [32]. The OR medication environment differs from that of an inpatient ward, and perioperative medication management often bypasses standard safety checks, such as electronic order entry with decision support, pharmacy approval of specific drugs before administration, and multiple nursing checks at administration [12]. Additionally, the medication environment of different hospitals (such as physical environment and human environment) has its particularity, which requires nurses to constantly summarise and explore medication experience in practice, strengthen their keen perception of risk factors in the medication environment, and then form the ability to adapt to their medication environment safety. Managers should create a supportive work environment of safe medication in the OR, promote nurses’ perception of their medication environment through regular department seminars, and encourage exchanges and learning among medical staff on medication safety experience to get familiar with possible risk factors in the medication environment and countermeasures.

#### 4.2.2 Working years.

It is worth noting that the results of multiple linear regression showed that the longer the working years, the higher the medication safety behaviour of OR nurses. This is consistent with the study by SLOSS et al., which argues that new nurses have a higher incidence of medication errors and are more likely to have unsafe or irregular behaviour in medication management [33]. In addition, relevant studies have shown that nurses who have worked for 5–10 years may be more likely to have unsafe drug use behaviour than nurses who have worked for 15–20 years [25] and newly graduated (less than 1 year of service) may have higher levels of medication insecurity than nurses with 1–5 years of service [26]. Drug management in the OR is difficult, and new nurses are not skilled in drug operation procedures, which may lead to blind implementation of medical orders. In addition, it may be that nurses with longer working years have more drug administration experience, and they are less susceptible to unsafe medication behaviours. In contrast, younger nurses are relatively less safety conscious and have drug administration experience. Therefore, nursing managers should focus on the drug safety ability of new nurses and train them in the knowledge and operation to make up for their lack of professional knowledge.

#### 4.2.3 Highest level of education.

Our studies showed that education is significantly positively correlated with medication-safe behaviour, consistent with HODKINSON et al. [8]. With the development of nursing education, nurses in tertiary hospitals gradually transition to higher education, the knowledge system of nursing education is constantly improved, and the training program of theoretical knowledge and clinical practice of nursing postgraduates goes hand in hand makes the medication safety ability of nursing undergraduates and postgraduates constantly improve. Therefore, young nurses should be encouraged to continue their education, and hospital managers should consider providing opportunities for continuing education based on merit.

#### 4.2.4 system and supervision.

*System and supervision* are important predictors of OR nurses’ safe medication behaviour. Ensuring strict implementation of a safe medication management system in OR is the core of ensuring safe medication for nurses. Previous studies have shown [26] that nurses have a lower score of unsafe medication behaviour when the department has a sound system and effective supervision. Boytim et al. [9] pointed out that haste, tension and progressive pressure were the high-frequency human factors leading to medication errors by nurses in the operating room. It can be seen that improving the medication safety behaviour of OR nurses is not only the behaviour of individual nurses but also should be included in the continuous improvement of the management system. Therefore, it is necessary to establish a perfect medication management system, such as the execution system of doctor’s orders, the drug administration process, and the patient’s drug safety monitoring system. Departments should also be called upon to regularly check the validity period and quality of drugs and reasonably arrange the level of nurses in each shift [34]. Additionally, Monitoring and reporting adverse drug events can ensure drug safety and improve medical quality, reflecting the degree of attention paid to drug safety in OR [35]. Relevant surveys showed that more than half of medication errors in the OR are not fully reported [36], and accurate identification and active reporting of medication errors by nurses are crucial to learning from mistakes and reducing similar errors. Therefore, an adverse drug event reporting system should be established to standardise the reporting process and disposal of adverse drug events, and surgeons, anesthesiologists, OR nurses, and other personnel should be encouraged to actively participate in Monitoring and reporting medication errors. According to a survey, clinical decision support algorithms can prevent 95% of self-reported medication errors in OR [37]. Information technology should be used to establish an electronic drug management system to realise the traceability of drug information and monitor and manage the medication process, which will identify and correct potential medication errors promptly.

#### 4.2.5 Professional title.

However, the higher the professional title, the worse the medication safety behaviour of the OR nurses. This result was similar to previous studies [25,26]. Although nurses with high professional titles have rich experience and are familiar with the complex clinical environment, they can still exhibit unsafe behaviours due to inertia thinking, set psychology, and temporary negligence. Moreover, nurses with high professional titles are mostly the department’s backbone and have multiple roles, making them more susceptible to external interference. In the future, more large-sample studies should be conducted on the group of operating room nurses to determine the impact of professional titles on their drug safety behaviours, and targeted training and education should be conducted for nurses with different professional titles.

#### 4.2.6 The last time medication safety training.

This study showed that the closer the training time is, the better the medication safety behaviour of the operating room nurses is. Some studies have found a strong correlation between healthcare workers who attend safety training seminars and increased rates of medication error reporting [38]. Time significantly impacts learning effectiveness, with memory degrading over time, completely forgotten when a threshold is reached, and learning effectiveness declining over time [39]. With the development of modern medical technology, drugs are updated rapidly, and the types are increasing year by year, which requires OR nurses to constantly understand the latest drug situation, master the principle, route, dosage, toxic and side effects of medication, to reduce the risk caused by adverse drugs. OR managers should regularly invite the heads of departments, anesthesiologists and pharmacists of the hospital to conduct training and lectures on medication safety knowledge so that OR nurses can improve their understanding of drug knowledge and new developments through drug training to reduce risks and ensure the safety of surgical patients.

### 4.1 Limitations

However, the study has some limitations. The nurses’ medication environment perception scale lacks the characteristics of OR specialities, and self-designed questionnaires are used to investigate the medication safety behaviour of OR nurses so that the results may be subjective. In the future, professional questionnaires or scientific objective evaluation tools with good reliability and validity and features of operating room specialities should be designed to further evaluate their medication safety behaviour. In addition, the hospitals in this study are all tertiary hospitals, and due to limited human and material resources, the sample size is small and comes from a specific region, which may limit the generality of the findings to other environments or populations. Future studies should conduct a multi-centre investigation to comprehensively explore the factors influencing the medication safety behaviour of OR nurses with different demographic characteristics.

## 5 Conclusion

In summary, the medication safety behaviour of nurses in the OR is at a low level. Medication environment, working years, the highest level of education, professional title, and the last time medication safety training influence medication safety behaviour. In the future, a supportive medication environment in OR should be created based on environmental factors such as department culture and system, relevant personnel and hardware facilities. The medication safety behaviour of OR nurses should be improved in various aspects, such as improving the drug safety management system, strengthening training and assessment, and establishing a safety reporting mechanism to reduce MAEs.

## References

[1] World Health Organization. WHO launches global effort to halve medication-related errors in 5 years. 2017 [cited 2024 Oct 20]. Available from: https://www.who.int/news/item/29-03-2017-who-launches-global-effort-to-halvemedication-related-errors-in-5-years

[2] Institute for Safe Medication Practices. Targeted Medication Safety Best Practices for Hospitals. 2022 [cited 2024 Dec 24]. Available from: https://www.ismp.org/guidelines/best-practices-hospitals

[3] Ryan A, Robertson K, Glass B. Look-alike medications in the perioperative setting: scoping review of medication incidents and risk reduction interventions. Int J Clin Pharm. 2024;46(1):26–39. 37688737 10.1007/s11096-023-01629-2 PMC10830657

[4] Smeulers M, Verweij L, Maaskant JM, de Boer M, Krediet CTP, Nieveen van Dijkum EJM, et al. Quality indicators for safe medication preparation and administration: a systematic review. PLoS One. 2015;10(4):e0122695. doi: 10.1371/journal.pone.0122695 25884623 PMC4401721

[5] Schroers G, Ross JG, Moriarty H. Nurses’ perceived causes of medication administration errors: a qualitative systematic review. Jt Comm J Qual Patient Saf. 2020. 10.1016/j.jcjq.2020.09.010 33153914

[6] Anugrahini CHRTS. Nurses′ compliance about patient safety in improving drug safety as an effort to reduce medication error:a literature review. Indones J Glob Health 2020:393–400.

[7] Di Simone E, Giannetta N, Spada E, Bruno I, Dionisi S, Chiarini M, et al. Prevention of medication errors during intravenous drug administration in intensive care units: a literature review. Recenti Prog Med. 2018;109(2):103–7. doi: 10.1701/2865.28902 29493633

[8] Suzuki R, Imai T, Sakai T, Tanabe K, Ohtsu F. Medication errors in the operating room: an analysis of contributing factors and related drugs in case reports from a japanese medication error database. J Patient Saf. 2022;18(2):e496–502. doi: 10.1097/PTS.0000000000000861 34009873

[9] Grissinger M. A loud wake-up call: unlabeled containers can lead to fatalities. P T. 2008;33(12):686–732. 19750047 PMC2730802

[10] Liu F, Lin P, Yu X. Reliability and validity of the nurse medication environmental perception scale. J. Nurs. Manag. 2018;18(06):386–90.

[11] Redman DD. Reducing medication errors in the OR. AORN J. 2017;105(1):106–9. doi: 10.1016/j.aorn.2016.11.007 28034386

[12] Nanji KC, Merry AF, Shaikh SD, Pagel C, Deng H, Wahr JA, et al. Global PRoMiSe (Perioperative Recommendations for Medication Safety): protocol for a mixed-methods study. BMJ Open. 2020;10(6):e038313. doi: 10.1136/bmjopen-2020-038313 32606066 PMC7328805

[13] Nanji KC, Patel A, Shaikh S, Seger DL, Bates DW. Evaluation of perioperative medication errors and adverse drug events. Anesthesiology. 2016;124(1):25–34. doi: 10.1097/ALN.0000000000000904 26501385 PMC4681677

[14] Kanjia M, Adler A, Buck D, Varughese A. Increasing compliance of safe medication administration in pediatric anesthesia by use of a standardised checklist. Paediatr Anaesth. 2019;29(3):258–64. 30609186 10.1111/pan.13578

[15] Hodkinson A, Tyler N, Ashcroft DM, Keers RN, Khan K, Phipps D, et al. Preventable medication harm across health care settings: a systematic review and meta-analysis. BMC Med. 2020;18(1):313. doi: 10.1186/s12916-020-01774-9 33153451 PMC7646069

[16] Yin L, Chen S, Yu J. Discussion on medication risk management for nurses in operating room. J Chin. Med. Administ. 2019;27(20):139–41.

[17] Wahr JA, Abernathy JH 3rd, Lazarra EH, Keebler JR, Wall MH, Lynch I, et al. Medication safety in the operating room: literature and expert-based recommendations. Br J Anaesth. 2017;118(1):32–43. doi: 10.1093/bja/aew379 28039240

[18] Mo L, Wu Z. Investigating risk factors for medication errors during perioperative care: A retrospective cohort study. Medicine (Baltimore). 2024;103(22):e38429. doi: 10.1097/MD.0000000000038429 39259066 PMC11142843

[19] Ye J. Patient safety of perioperative medication through the lens of digital health and artificial intelligence. JMIR Perioper Med 2023;6:e34453. 37256663 10.2196/34453 PMC10267793

[20] von Elm E, Altman DG, Egger M, Pocock SJ, Gøtzsche PC, Vandenbroucke JP, et al. The Strengthening the Reporting of Observational Studies in Epidemiology (STROBE) statement: guidelines for reporting observational studies. Lancet. 2007;370(9596):1453–7. doi: 10.1016/S0140-6736(07)61602-X 18064739

[21] Chen X, Yue L, Li B, Li J, Wu X, Peng B, et al. Status and related factors of professional growth among young nursing talents: a cross-sectional study in China. BMC Nurs. 2024;23(1):116. doi: 10.1186/s12912-024-01790-7 38360608 PMC10870662

[22] Li Z-Y, Cao X, Li S, Huang T-J, Liu Y-X, Qin L-H. Spiritual needs and influencing factors among people with stroke in China: a cross-sectional study. BMC Nurs. 2024;23(1):507. doi: 10.1186/s12912-024-02182-7 39075439 PMC11287944

[23] Chen X, Tao Z, Tang Y, Yan X. Status and associations of nursing practice environments in intensive care units: A cross-sectional study in China. J Nurs Manag. 2022;30(7):2897–905. doi: 10.1111/jonm.13616 35403326

[24] Jafaru Y, Abubakar D. Medication administration safety practices and perceived barriers among nurses: a cross-sectional study in Northern Nigeria. Glob J Qual Saf Healthc. 2022;5(1):10–7. doi: 10.36401/JQSH-21-11 37260556 PMC10229023

[25] Qin N, Shi S, Duan Y, Zhong Z, Xiang G. Self-reported unsafe medication behaviour among clinical nurses in China: A nationwide survey. Nurs Open. 2023;10(2):1060–70. doi: 10.1002/nop2.1373 36177807 PMC9834539

[26] Yu X, Li C, Gao X, Liu F, Lin P. Influence of the medication environment on the unsafe medication behaviour of nurses: A path analysis. J Clin Nurs. 2018;27(15–16):2993–3000. doi: insert_doi_here 29679412 10.1111/jocn.14485

[27] Daigle S, Talbot F, French DJ. Mindfulness-based stress reduction training yields improvements in well-being and rates of perceived nursing errors among hospital nurses. J Adv Nurs. 2018;74(10):2427–30. doi: 10.1111/jan.13729 29869350

[28] Reason J. Understanding adverse events: human factors. Qual Health Care. 1995;4(2):80–9. doi: 10.1136/qshc.4.2.80 10151618 PMC1055294

[29] Molloy GJ, O’Boyle CA. The SHEL model: a useful tool for analyzing and teaching the contribution of Human Factors to medical error. Acad Med. 2005;80(2):152–5. doi: 10.1097/00001888-200502000-00009 15671319

[30] Bakhshi F, Mitchell R, Nasrabadi AN, Varaei S, Hajimaghsoudi M. Behavioural changes in medication safety: Consequent to an action research intervention. J Nurs Manag. 2021;29(2):152–64. doi: 10.1111/jonm.13128 32955774

[31] Olds DM, Aiken LH, Cimiotti JP, Lake ET. Association of nurse work environment and safety climate on patient mortality: A cross-sectional study. Int J Nurs Stud. 2017;74:155–61. doi: 10.1016/j.ijnurstu.2017.06.004 28709013 PMC5695880

[32] Ding YY. Research on improving the happiness of work based on the theory of mutual settlement. Chines Foreig Entrepren. 2015;28:56–7. doi: 10.3969/j.issn.1000-8772.2015.28.020

[33] Sloss EA, Jones TL, Baker K, Robins J, Thacker LR. Describing medication administration and alert patterns experienced by new graduate nurses during the first year of practice. Comput Inform Nurs 2024;42(2):94–103. 38062552 10.1097/CIN.0000000000001035

[34] Mahmood A, Chaudhury H, Valente M. Nurses’ perceptions of how physical environment affects medication errors in acute care settings. Appl Nurs Res. 2011;24(4):229–37. doi: 10.1016/j.apnr.2009.08.005 22099470

[35] Whitaker D, Brattebø G, Trenkler S, Vanags I, Petrini F, Aykac Z, et al. The European board of anaesthesiology recommendations for safe medication practice: first update. Eur J Anaesthesiol. 2017;34(1):4–7. doi: 10.1097/EJA.0000000000000531 27548778

[36] Rizalar S, Topcu SY. The patient safety culture perception of Turkish nurses who work in operating room and intensive care unit. Pak J Med Sci. 2017;33(2):374–9. doi: 10.12669/pjms.332.11727 28523040 PMC5432707

[37] Amici LD, van Pelt M, Mylott L, Langlieb M, Nanji KC. Clinical decision support as a prevention tool for medication errors in the operating room: a retrospective cross-sectional study. Anesth Analg. 2024;139(4):832–9. doi: 10.1213/ANE.0000000000007058 38870073

[38] Gleeson L, Dalton K, O’Mahony D, Byrne S. Interventions to improve reporting of medication errors in hospitals: A systematic review and narrative synthesis. Res Social Adm Pharm. 2020;16(8):1017–25. doi: 10.1016/j.sapharm.2019.12.005 31866121

[39] Georgiou A, Katkov M, Tsodyks M. Retroactive interference model of forgetting. J Math Neurosci. 2021;11(1):4. doi: 10.1186/s13408-021-00102-6 33484358 PMC7826326

