# Supplementary material for: Status and related factors of medication safety behaviour of nurses in the operating room: A cross-sectional survey in China
Source: PLoS One. 2025 Apr 28;20(4):e0320264. doi: 10.1371/journal.pone.0320264 (PMC12036857; doi:10.1371/journal.pone.0320264)
Supplement: Attachment — Submitted filename: Response to Reviewers.docx [file pone.0320264.s002.docx]

**PONE-D-24-50882**

**Status and Related Factors of Medication Safety Behavior of Nurses in Operating Room: A Cross-Sectional Survey in China**

**PLOS ONE**

Dear Editor-in-Chief,

Thank you very much for your careful review and constructive suggestions with regard to our manuscript **“Status and Related Factors of Medication Safety Behavior of Nurses in Operating Room: A Cross-Sectional Survey in China”.**

We have carefully evaluated all the comments and suggestions, and responded to them in our revised manuscript accordingly. All the authors agreed upon and approved the contents of the manuscript. Again, we appreciate the scholarly work by the Editors/Reviewers, and hope our detailed revisions have addressed all the criticisms raised by the reviewers.

Please contact us with any further questions and we are looking forward to your positive consideration.

Sincerely,

Xiuwen Chen

Teaching and Research Section of Clinical Nursing

Xiangya Hospital

Central South University
Changsha, 410008, China

chenxiuwen2020@163.com

Please include the following items when submitting your revised manuscript:

A rebuttal letter that responds to each point raised by the academic editor and reviewer(s). You should upload this letter as a separate file labeled 'Response to Reviewers'.

**Answer:** Thank you for your suggestion.

We have included a separate "Response to Reviewers" document addressing each point raised.

A marked-up copy of your manuscript that highlights changes made to the original version. You should upload this as a separate file labeled 'Revised Manuscript with Track Changes'.

**Answer:** Thank you for your reminder.

We have provided a marked-up copy of the manuscript labeled **'Revised Manuscript with Track Changes'** for your review.

An unmarked version of your revised paper without tracked changes. You should upload this as a separate file labeled 'Manuscript'.

**Answer:** Thank you for your reminder.

We have provided an unmarked version of the revised paper labeled **'Manuscript'** for your review.

If you would like to make changes to your financial disclosure, please include your updated statement in your cover letter. Guidelines for resubmitting your figure files are available below the reviewer comments at the end of this letter.

**Answer:** Thank you for your notice.

We do not need to make any changes to our financial disclosure. In addition, we have followed the guidelines provided for resubmitting the figure files.

If applicable, we recommend that you deposit your laboratory protocols in protocols.io to enhance the reproducibility of your results. Protocols.io assigns your protocol its own identifier (DOI) so that it can be cited independently in the future. For instructions see: https://journals.plos.org/plosone/s/submission-guidelines#loc-laboratory-protocols. Additionally, PLOS ONE offers an option for publishing peer-reviewed Lab Protocol articles, which describe protocols hosted on protocols.io. Read more information on sharing protocols at <https://plos.org/protocols?utm_medium=editorial-email&utm_source=authorletters&utm_campaign=protocols.>

**Answer:** Thank you for the thoughtful suggestion.

Our team carefully considered your recommendation. However, as we have provided a detailed description of our methods in the manuscript, we are not planning to deposit our laboratory protocols in protocols.io at this time. Nonetheless, we sincerely appreciate the suggestion and will keep it in mind for future projects.

Journal Requirements:

1. When submitting your revision, we need you to address these additional requirements.

Please ensure that your manuscript meets PLOS ONE's style requirements, including those for file naming. The PLOS ONE style templates can be found at
[https://journals.plos.org/plosone/s/file?id=wjVg/PLOSOne_formatting_sample_main_body.pdf](https://track.editorialmanager.com/CL0/https:%2F%2Fjournals.plos.org%2Fplosone%2Fs%2Ffile%3Fid=wjVg%2FPLOSOne_formatting_sample_main_body.pdf/1/010f0193d7653da7-5af1b3be-ca04-4e86-83c9-af444a5e9237-000000/zDm59Oi3QjjnKrReHcf_ySLoi6Z-m9amXOBphWA1KCI=189) and
[https://journals.plos.org/plosone/s/file?id=ba62/PLOSOne_formatting_sample_title_authors_affiliations.pdf](https://track.editorialmanager.com/CL0/https:%2F%2Fjournals.plos.org%2Fplosone%2Fs%2Ffile%3Fid=ba62%2FPLOSOne_formatting_sample_title_authors_affiliations.pdf/1/010f0193d7653da7-5af1b3be-ca04-4e86-83c9-af444a5e9237-000000/lv18rRxiXGip4bRyG2goQ-knIqF-T8cyySB73zzhPJ4=189)
**Answer:** Thank you for the suggestion.

We have carefully reviewed the PLOS ONE's style requirements and ensured that both our manuscript and file names fully comply with the provided guidelines.

2. Please provide additional details regarding participant consent. In the ethics statement in the Methods and online submission information, please ensure that you have specified (1) whether consent was informed and (2) what type you obtained (for instance, written or verbal, and if verbal, how it was documented and witnessed). If your study included minors, state whether you obtained consent from parents or guardians. If the need for consent was waived by the ethics committee, please include this information.

If you are reporting a retrospective study of medical records or archived samples, please ensure that you have discussed whether all data were fully anonymized before you accessed them and/or whether the IRB or ethics committee waived the requirement for informed consent. If patients provided informed written consent to have data from their medical records used in research, please include this information.
**Answer:** Thank you for the suggestion.

In section 2.5 Ethical considerations, we showed that the study was approved by the Ethics Committee of Xiangya Hospital, Central South University (registry number 2022100085). The study complied with the Declaration of Helsinki and all participants signed informed consent forms and their participation was anonymous. We have also uploaded the attachment of the corresponding ethical approval certificate.

3. Please ensure that you have specified a) Did participants provide their written or verbal informed consent to participate in this study?
b) If consent was verbal, please explain i) why written consent was not obtained, ii) how you documented participant consent, and iii) whether the ethics committees/IRB approved this consent procedure."
**Answer:** Thank you for the suggestion.

We have ensured the participants provide their written informed consent to participate in this study.

- In consent please state in Ethics Method section and manuscript if it is written or verbal. If consent was verbal, please explain a) why written consent was not obtained, b) how you documented participant consent, and c) whether the ethics committees/IRB approved this consent procedure.
**Answer:** Thank you for your careful feedback

In section 2.5 Ethical considerations, we showed that all participants signed informed consent forms and their participation was anonymous.

4. Thank you for stating the following financial disclosure:
[This work was supported by the 2024 Annual Graduate Student Independent Exploration and Innovation Project of Central South University (2024ZZTS0548)].

Please state what role the funders took in the study. If the funders had no role, please state: ""The funders had no role in study design, data collection and analysis, decision to publish, or preparation of the manuscript.""
**Answer:** Thank you for your reminder.

We have clarified in the cover letter that the funders provided support for the material printing cost during the questionnaire collection and the layout cost during the later publication of the article but had no role in study design, data analysis, decision to publish, or preparation of the manuscript.

If this statement is not correct you must amend it as needed.

Please include this amended Role of Funder statement in your cover letter; we will change the online submission form on your behalf.
**Answer:** Thank you for the suggestion.

The statement is correct.

5. In the online submission form, you indicated that The data that support the findings of this study are available on request from the corresponding].

All PLOS journals now require all data underlying the findings described in their manuscript to be freely available to other researchers, either 1. In a public repository, 2. Within the manuscript itself, or 3. Uploaded as supplementary information.

This policy applies to all data except where public deposition would breach compliance with the protocol approved by your research ethics board. If your data cannot be made publicly available for ethical or legal reasons (e.g., public availability would compromise patient privacy), please explain your reasons on resubmission and your exemption request will be escalated for approval.
**Answer:** Thank you for your careful feedback

We have clearly described the methods of all data generated or analysed during this study in our manuscript, and we have also uploaded the raw data.

6. PLOS requires an ORCID iD for the corresponding author in Editorial Manager on papers submitted after December 6th, 2016. Please ensure that you have an ORCID iD and that it is validated in Editorial Manager. To do this, go to ‘Update my Information’ (in the upper left-hand corner of the main menu), and click on the Fetch/Validate link next to the ORCID field. This will take you to the ORCID site and allow you to create a new iD or authenticate a pre-existing iD in Editorial Manager.

**Answer:** Thank you for your guidance.

We have confirmed that the corresponding author has an ORCID iD of 0000-0001-7349-3588, and we have followed your instructions to ensure that it is verified in Editorial Manager.

**Additional Editor Comments:**

References and Content Clarification:
- Conduct a thorough revision of the English language to address grammatical and stylistic errors. Consider professional editing services.

**Answer:** Thank you for your careful feedback

We read thorough the whole text again and have made revisions based on your feedback and sought the assistance of a professional service for language editing to improve clarity and correctness.

- Ensure the study's objectives are clearly addressed in the abstract.

**Answer:** Thank you for your valuable guidance.

We have carefully reviewed the abstract and have made revisions to the aim and results sections in line with your suggestion to ensure the study's objectives are clearly addressed in the abstract.

-Replace Reference 9 (Cooper et al., 1984) as it is outdated and pertains to anesthesiologists rather than operating room nurses. Ensure all references are current and relevant to the context of OR nurses in China.

**Answer:** Thank you for the thoughtful suggestion.

We have deleted Reference 9 (Cooper et al., 1984) and rewritten the second paragraph of the Introduction. We have reviewed the references in our manuscript once again and confirmed that all references are current and relevant to the context of OR nurses in China.

Address the findings by Song et al. (2022) regarding the most common medical errors in operating room settings (e.g., surgical instruments, sterilization) and clarify the relevance of medication errors to your study.

**Answer:** Thank you for the thoughtful suggestion.

We have carefully read the article by Song et al. Although Song et al reported that medication errors are not the most common medical errors of operating room nurses, compared with other departments, there are many high-risk drugs in operating rooms, and management is difficult. Medication errors will bring serious harm to patients, and most medication errors are preventable. Therefore, it is very necessary to explore the problem of medication errors in the operating room. In addition, we have reorganized the logic of the Introduction with reference to your valuable comments, and focused this part(Line 55-72 in the revised manuscript with track changes) on the harm of medication errors in the operating room to highlight the necessity of our research.

Thank you again for your valuable feedback. If you have any further questions or concerns, please feel free to reach out to us.

Unreferenced Sections (Lines 67-76):
- Add appropriate references to substantiate claims, particularly those involving medication safety practices.

**Answer:** Thank you for your reminder.

We have added appropriate references to substantiate claims and rewritten the second paragraph of the introduction(Line 55-72 in the revised manuscript with track changes).

Clarify whether administration of anesthetic drugs is performed by nurses in the study setting.

**Answer:** Thank you for your reminder.

We have refined this part to make it more clear(Line 74-79 in the revised manuscript with track changes) and add references. Operating room nurses play a crucial role in the drug administration process, mainly including dispensing drugs, checking drugs, and assisting the anesthesiologist to administer drugs. The operating room nurse serves as an assistant to assist the anesthesiologist in administering anesthetic drugs and performing appropriate intraoperative administration as instructed by the doctor.

- Sample Size and Variables: Clearly justify the sample size and explain how it is sufficient for the study's 11 variables.

**Answer:** Thank you for your notice.

First, we sincerely apologize for the error. To ensure data accuracy, our team carefully double-checked all the variables. Unfortunately, we discovered an error in the variables of the original manuscript. But we have revised it at the section 2.2 “Study Design and participants” and it should be 15 independent variables (10 demographic variables, five dimensions of nurses' medication environment perception scale). We hope this clarifies the situation, and we appreciate your understanding. If you have any further questions or concerns, please feel free to reach out to us.

- Provide details on the sampling technique used.

**Answer:** Thank you for your reminder.

We have provided details on the sampling technique used. In response to your suggestions, we have made the necessary additions at the section 2.1 “Study Design and participants” in the revised manuscript with track changes. We have provided details on the sampling techniques and added specifics to the sample size calculation (10 demographic variables, five dimensions of nurses' medication environment perception scale) .

- Ethical Considerations: Clarify whether informed consent was obtained from all study participants.

**Answer:** Thank you for your notice.

we added that “all participants signed informed consent” at the section of 2.5 “Ethical considerations” of line 157 in the revised manuscript with track changes.

- Include confidence intervals and p-values for all statistically significant results.

**Answer:** Thank you for your careful feedback

We have added *p*-values in the results section of abstract.

- Expand the discussion to provide deeper insights into your findings and align them with relevant current literature.

**Answer:** Thank you for your valuable feedback.

We have expanded the discussion section after carefully reading the full text and related references to provide deeper insights into our findings and align them with relevant current literature. Additionally, for the discussion, we use subheadings to make it more explicit and cite a large number of references to support our findings.

- Practical Significance: Reassess the practical significance of your findings given the sample size limitations. Discuss any implications for real-world applications.

**Answer:** Thank you for your careful feedback.

We have reassessed the practical significance of our findings given the sample size limitations. In the discussion part, a large number of references are cited as evidence, and the limitations of the sample size are explained again.

- Review the manuscript carefully to ensure adherence to PLOS ONE's formatting and writing guidelines.

**Answer:** Thank you for your reminder.

We have carefully reviewed the PLOS ONE style requirements and ensured that both our manuscript and file names fully comply with the provided guidelines.

Best of luck in your revision.


[Note: HTML markup is below. Please do not edit.]

Reviewers' comments:

Reviewer's Responses to Questions

**Comments to the Author**

1. Is the manuscript technically sound, and do the data support the conclusions?

The manuscript must describe a technically sound piece of scientific research with data that supports the conclusions. Experiments must have been conducted rigorously, with appropriate controls, replication, and sample sizes. The conclusions must be drawn appropriately based on the data presented.

Reviewer #1: No

**Answer:** Thank you for your valuable feedback.

We have made comprehensive revisions based on the editor's and reviewers' comments to ensure that experiments are conducted rigorously. We have also modified and supplemented the sampling method and sample size calculation as suggested.  We also discussed and explained the limitations of the sample size, and re-evaluated the real-world implications of our findings.

Additionally, our team has rechecked and analyzed the data once more and consulted a statistical expert to verify the accuracy of our data analysis methods.

Reviewer #2: Partly

**Answer:** Thank you for your review.

We have made comprehensive revisions based on the editor's and reviewers' comments to ensure that experiments are conducted rigorously. We have also modified and supplemented the sampling method and sample size calculation as suggested.  We also discussed and explained the limitations of the sample size, and re-evaluated the real-world implications of our findings.

Additionally, our team has rechecked and analyzed the data once more and consulted a statistical expert to verify the accuracy of our data analysis methods.

2. Has the statistical analysis been performed appropriately and rigorously?

Reviewer #1: No

**Answer:** Thank you for your valuable feedback.

We have made comprehensive revisions based on the editor's and reviewers' comments to ensure that the statistical analysis has been performed appropriately and rigorously. Additionally, our team has rechecked and analyzed the data once more and consulted a statistical expert to verify the accuracy of our data analysis methods.

Reviewer #2: Yes

**Answer:** Thank you for your positive feedback.

3. Have the authors made all data underlying the findings in their manuscript fully available?

The [PLOS Data policy](http://track.editorialmanager.com/CL0/http:%2F%2Fwww.plosone.org%2Fstatic%2Fpolicies.action%23sharing/1/010f0193d7653da7-5af1b3be-ca04-4e86-83c9-af444a5e9237-000000/H-XxXAvwwOsZfMPkfBvhfGkyPk_MQqu5DQpNkCjUiGU=189) requires authors to make all data underlying the findings described in their manuscript fully available without restriction, with rare exception (please refer to the Data Availability Statement in the manuscript PDF file). The data should be provided as part of the manuscript or its supporting information, or deposited to a public repository. For example, in addition to summary statistics, the data points behind means, medians and variance measures should be available. If there are restrictions on publicly sharing data—e.g. participant privacy or use of data from a third party—those must be specified.

Reviewer #1: Yes

**Answer:** Thank you for your positive feedback.

Reviewer #2: Yes

**Answer:** Thank you for your positive feedback.

4. Is the manuscript presented in an intelligible fashion and written in standard English?

PLOS ONE does not copyedit accepted manuscripts, so the language in submitted articles must be clear, correct, and unambiguous. Any typographical or grammatical errors should be corrected at revision, so please note any specific errors here.

Reviewer #1: No

**Answer:** Thank you for highlighting this important point.

We have made revisions based on your feedback and sought the assistance of a professional service for language editing to improve clarity and correctness. We appreciate your guidance and welcome any further concerns you may have.

Reviewer #2: Yes

**Answer:** Thank you for your positive feedback.

5. Review Comments to the Author

Please use the space provided to explain your answers to the questions above. You may also include additional comments for the author, including concerns about dual publication, research ethics, or publication ethics. (Please upload your review as an attachment if it exceeds 20,000 characters)

**Answer:** Thank you for your thorough review and comments.

We have carefully considered your feedback and made the necessary revisions to address the issues raised. We sincerely appreciate your attention to detail and guidance in improving the manuscript. If you have any further concerns or additional comments, please do not hesitate to let us know.

Reviewer #1: Review Comments
Line 56-59: The content mentioned in Reference 9 states that anesthesiologists are responsible for single use of drugs in the operating room, without a double-checking step. The following points need clarification:

The content in Reference 9 (Cooper JB, Newbower RS, Kitz RJ. An analysis of major errors and equipment failures in anesthesia management: considerations for prevention and detection. Anesthesiology 1984; 60: 34–42) is based on a 1984 study. In China, as of 2024, it is rare for anesthesiologists to administer drugs without double-checking; almost all perform double checks.
**Answer:** Thank you for your valuable feedback.

We fully understand your concern regarding Reference 9 (Cooper JB, Newbower RS, Kitz RJ. An analysis of major errors and equipment failures in anesthesia management: considerations for prevention and detection. Anesthesiology 1984; 60: 34–42). After carefully considering your comments, we have decided to delete this reference and the content of the citation from our manuscript.  Additionally, we have rewritten the second paragraph of the introduction(Line 55-72 in the revised manuscript with track changes)

The study subjects in Reference 9 were anesthesiologists, not operating room nurses, who are the subjects of this article. Recent investigations into medical errors among operating room nurses (Song Q, Tang J, Wei Z, Sun L. Prevalence and associated factors of self-reported medical errors and adverse events among operating room nurses in China. Front Public Health. 2022;10:988134. doi: 10.3389/fpubh.2022.988134) show that the most common medical errors stem from surgical instruments (9.1%), sterilization (9.0%), equipment and consumables (8.9%), and specimen management (7.8%). Thus, medication errors are not among the most common medical errors in the operating room.
**Answer:** Thank you for your careful review.

We appreciate your pointing out that the study subjects in Reference 9 were anesthesiologists, not operating room nurses, who are the focus of our article. After carefully reading Reference 9(Cooper JB, Newbower RS, Kitz RJ. An analysis of major errors and equipment failures in anesthesia management: considerations for prevention and detection. Anesthesiology 1984; 60: 34–42), we decided to delete the reference and the quoted content.

Additionally, we acknowledge the recent investigations into medical errors among operating room nurses, as highlighted by the study by Song et al. (2022), which show that the most common medical errors in the operating room stem from surgical instruments, sterilization, equipment and consumables, and specimen management, rather than medication errors. We have carefully read the article. Although Song et al reported that medication errors are not the most common medical errors of operating room nurses, compared with other departments, there are many high-risk drugs in operating rooms, and management is difficult. Medication errors will bring serious harm to patients, and most medication errors are preventable. Therefore, it is very necessary to explore the problem of medication errors in the operating room. In addition, we have reorganized the logic of the Introduction with reference to your valuable comments, and focused this part(Line 55-73 in the revised manuscript with track changes) on the harm of medication errors in the operating room to highlight the necessity of our research.

Thank you again for your valuable feedback. If you have any further questions or concerns, please feel free to reach out to us.

Line 67-71: There is no reference to support this section. Considering the previous discussion pertains to anesthesiologists rather than operating room nurses, I suspect the author intended to refer to MAEs caused by anesthesiologists rather than operating room nurses. This leads to a lack of basis for this study and suggests that the author should review this section.
**Answer:** Thank you for your insightful suggestions.

We have read this section carefully and reworked the logic. After discussion we have decided to delete Line 67-71 in order to avoid controversy. We have rewritten the second paragraph of the introduction(Line 55-72 in the revised manuscript with track changes), and focused this part on the harm of medication errors in the operating room. After introducing the harm of medication errors in the operating room, we then described the important role of the operating room nurses in the medication process, so as to introduce the importance of improving the safe medication behavior of the operating room nurses, thus highlighting our research theme.

Line 72-76: There is no reference to support this section. It is unclear whether the administration of anesthetic drugs is performed by nurses.
**Answer:** Thank you for raising this point.

We understand your concerns about the lack of reference support for this section and the uncertainty regarding the role of nurses in administering anesthetic drugs. Firstly, regarding the lack of references, we acknowledge that we omitted relevant literature in our previous version.

Line 73-77 in the revised manuscript with track changes: We added reference 8 (Suzuki R, Imai T, Sakai T, Tanabe K, Ohtsu F. Medication Errors in the Operating Room: An Analysis of Contributing Factors and Related Drugs in Case Reports from a Japanese Medication Error Database. J Patient Saf 2022;18(2):e496-e502) and reference 16 (Yin L, Chen S, Yu J. Discussion on medication risk management for nurses in operating room. Journal of Chinese medicine administration 2019;27(20):139-141).

We have also refined this part to make it more clear. Operating room nurses play a crucial role in the drug administration process, mainly including dispensing drugs, checking drugs, and assisting the anesthesiologist to administer drugs. The operating room nurse serves as an assistant to assist the anesthesiologist in administering anesthetic drugs and performing appropriate intraoperative administration as instructed by the doctor.

Line 95: The study's 11 variables do not match the research variable count, indicating an insufficient sample size.
**Answer:** Thank you for your careful review.

First, we sincerely apologize for the error. To ensure data accuracy, our team carefully double-checked all the variables. Unfortunately, we discovered an error in the variables of the original manuscript. But we have revisioned it at the section 2.1 “Study Design and participants” and it should be 15 independent variables (10 demographic variables, five dimensions of nurses' medication environment perception scale). We have added specifics to the 15 variables.

For the sample size, the recommendations of between 5 and 10 times the number of independent variables and considering that 20% of invalid questionnaires, a minimum of 90 operating nurses was required. We hope this clarifies the situation, and we appreciate your understanding. If you have any further questions or concerns, please feel free to reach out to us.

Informed Consent: It is unclear whether the study subjects signed informed consent forms.
**Answer:** Thank you for raising this point.

Upon carefully examining your comments, we realize that there may have been some misunderstanding or unclear statement in our description of manuscript.

We described the informed consent of study subjects in the original manuscript. For example，Line 152-153: The study complied with the Declaration of Helsinki and all participants gave informed consent and their participation was anonymous.

Line91-92: (4) informed consent and voluntary participation in the study.

In addition, our previous descriptions may not have been clear enough, in reponse to your suggestion, we added that “all participants signed informed consent” at the section of 2.5 “Ethical considerations” of line 157 in the revised manuscript with track changes.

Correlation Analysis Results: Even if the correlation analysis results are significant, the small sample size means that the observed associations have minimal practical significance in real-world applications.

**Answer:** Thank you for your valuable feedback.

Your point is well-taken, and we acknowledge that the small sample size may limit the generality of our findings in real-world applications. The small sample size is a major limitation of our article. Although the correlation analysis results are significant, they still need to be interpreted with caution. We have reassessed the practical significance of our findings given the sample size limitations. In the discussion part, a large number of references are cited as evidence and the sample size problem is further explained, so that readers can carefully consider our research results. We also discuss this in the section Limitations of the revised manuscript. To enhance the practical significance of our research, we plan to expand the sample size in future studies and validate our findings in different populations and settings. This will help to more comprehensively assess the relationships between variables and provide stronger evidence for their effectiveness in real-world applications. Once again, thank you for your valuable comments. We have taken your suggestions into consideration and will strive to improve our research in subsequent studies.

Reviewer #2: Dear author/s,
The manuscript had the aim to explore the current status and related factors regarding medication safety behavior of operating room nurses in China. The study is important; however, the study has some limitations.
Abstract
The objective of this study was to explore the current status and related factors regarding medication safety behavior of operating room nurses. But, in my opinion, the objectives of this study are not answered well.
Result: It is better to write with confidence intervals and/or p-values of those listed as statistically significant factors.

**Answer:** Thank you for your valuable feedback

We have carefully reviewed the abstract and have made revisions to the aim and results sections in line with your suggestion to ensure the study's objectives are clearly addressed in the abstract.

Additionally, we have added p-values in the result section of abstract.

Keywords: Please write the keywords based on MeSH terms.

**Answer:** Thank you for your valuable feedback

We have searched the Mesh terms thesaurus and reformulated the keywords to ensure that all the keywords are Mesh terms.

I believe that a revision of the English language is necessary because there are a lot of grammatical errors throughout the document.
**Answer:** Thank you for highlighting this important point.

We have made revisions based on your feedback and sought the assistance of a professional service for language editing to improve clarity and correctness. We appreciate your guidance and welcome any further concerns you may have.

The authors should consider a review of cited data and follow the PlOS ONE journal authors writing guidelines.

**Answer:**Thank you for your suggestion.

We have carefully reviewed our cited data to ensure its completeness and accuracy. We can confirm that no retracted papers have been cited in this manuscript. All referenced literature is valid, relevant, and supports our research viewpoints and conclusions.

We have carefully reviewed the PLOS ONE style requirements and ensured that both our manuscript and file names fully comply with the provided guidelines.

The authors do not write with clear sample size calculation and sampling techniques. Please clarify that.

**Answer:** Thank you for your valuable suggestion.

We understand your concern and appreciate your guidance in improving our manuscript. In response to your suggestions, we have made the necessary additions at the section 2.1 “Study Design and participants” in the revised manuscript with track changes. We have provided details on the sampling techniques and added specifics to the sample size calculation (10 demographic variables, five dimensions of nurses' medication environment perception scale) .

The result of this study needs revision.

**Answer:** Thank you for your careful review.

We have revised the results according to the article guidelines to ensure compliance with journal requirements. we fully understand and acknowledge the importance of ensuring the accuracy and robustness of our findings. In response to this feedback, our team carefully double-checked all the original data and re-analyzed the data to ensure the reliability of our results. Additionally, for your convenience, we have included the raw data (labeled as "**Additional File 1: The Raw Data**"). If you have any further questions or concerns, please feel free to reach out to us.

The discussion is poor. The findings could have been better discussed.

**Answer:** Thank you for your thorough review and comments.

You are correct that the discussion of our findings could have been more comprehensive and insightful. In the revised version of the manuscript, we have made significant improvements to the discussion section to better interpret and contextualize our results.

After carefully reading the full text and related references, we have expanded the discussion section to provide deeper insights into our findings and align them with relevant current literature. Additionally, for the discussion, we use subheadings to make it more explicit and cite a large number of literatures to support our findings. We apologize for any shortcomings in the previous version of the manuscript and appreciate your guidance in improving our work. Thank you once again for your valuable feedback. We are committed to continuously refining our manuscript to ensure that it is as robust and insightful as possible.
